# Mid-Holocene aridity recorded in pygmy hippo and giant tortoise bone from southwest Madagascar

**DOI:** 10.1098/rspb.2025.0493

**Published:** 2025-05-21

**Authors:** Sean Hixon

**Affiliations:** ^1^ Integrative Biology, Oregon State University, Corvallis, OR, USA; ^2^ Max Planck Institute for Geoanthropology, Jena, Germany

**Keywords:** palaeoecology, palaeoclimate, aridification, radiocarbon, stable isotope, extinction

## Abstract

Aridity can exacerbate threats to endemic biodiversity, and arid intervals during the last couple of millennia may have contributed to endemic large herbivore extinctions on Madagascar. However, regional palaeoclimate records spanning multiple millennia are limited, and the tolerance of extinct taxa to past water scarcity is poorly known. To infer changes in the diet and habitat aridity of extinct pygmy hippos and giant tortoises during approximately 6000–1000 years ago, I used carbon and nitrogen isotope (*δ*^13^C and *δ*^15^N) data from 49 directly radiocarbon-dated bones collected around Tampolove, southwest Madagascar. Fluctuations in bone *δ*^15^N values through time in both species indicate tolerance of dry habitat during intermittent drying trends, including around a dry period known as the ‘4.2 ka event’. However, taxon-specific differences in the covariance of bone *δ*^13^C and *δ*^15^N values suggest that the diets of pygmy hippos and giant tortoises changed in different ways during these past arid intervals. This suggests that past aridification had different effects on these taxa. Thus, I argue that hypotheses for past extinction that involve a synergy among climate drying and forest clearance, hunting and biological invasion must consider taxon-specific responses to past aridity.

## 1. Introduction

Madagascar’s diversity of microendemic organisms follows from millions of years of allopatric speciation [1], is actively being inventoried [2] and faces multiple threats today [3]. Yet these threats are not all unique to modern times; a suite of enduring threats (hunting, introduced predators and competitors, forest clearance and climate change) coincided with multiple endemic vertebrate extinctions within the past millennium [4–10]. Although temporal coincidence is an essential starting point for identifying plausible explanations of past species losses, synergy hypotheses for past extinction are complicated by the direct and indirect ways in which stressors interact. This is particularly clear in the case of water scarcity, which affects organisms directly, as well as indirectly through its effects on populations of potential antagonists [11,12]. To start to effectively test synergy hypotheses for extinction that involve water scarcity, it is necessary to compare the direct effects of water scarcity on different taxa [13], which can be done back through time using the fossil record. Here, I present such a comparison between extinct pygmy hippos (*Hippopotamus* spp.) and giant tortoises (*Aldabrachelys* spp.) by using multi-millennial records of stable isotope values from directly radiocarbon-dated bone collected around Tampolove, southwest Madagascar.

Taxon-specific sensitivity to water scarcity leaves an identifiable signature at multiple spatial scales. Regionally, simultaneous changes in climate and shifts in a species’ geographical range over centuries and millennia can reflect sensitivity to environmental change [14,15]. Most of Madagascar’s subfossil sites are concentrated in the arid southwest [10]. However, traces of past geographical ranges and range dynamics can persist in a species phylogeography and in the demographic history of extant populations [16] as well as in the temporal distribution of subfossil bone from particular sites [17,18].

Locally, sensitivity to water scarcity can be inferred from decoupled habitat aridity (i.e. habitat that an animal experiences) and regional aridity (i.e. prevailing conditions in a region, in the sense of [19]). For example, populations of animals sensitive to water scarcity (e.g. *Hippopotamuses*) may move across a landscape over time to persistently exploit wet patches of preferred browse in the face of a regional drying trend. This persistent preference (i.e. conserved niche) can be recognized in the comparatively invariant stable isotope content of the animals’ bones [20]. Conversely, taxa insensitive to water scarcity may exploit the prevalent habitat, with little behavioural filter (*sensu* [21]), thus effectively tracking regional environmental changes and leaving a record of biological remains that is coupled with regional aridity. In such cases (e.g. [22,23]), the animals’ stable isotope record can be interpreted primarily in terms of past environmental change.

**Figure 1.**
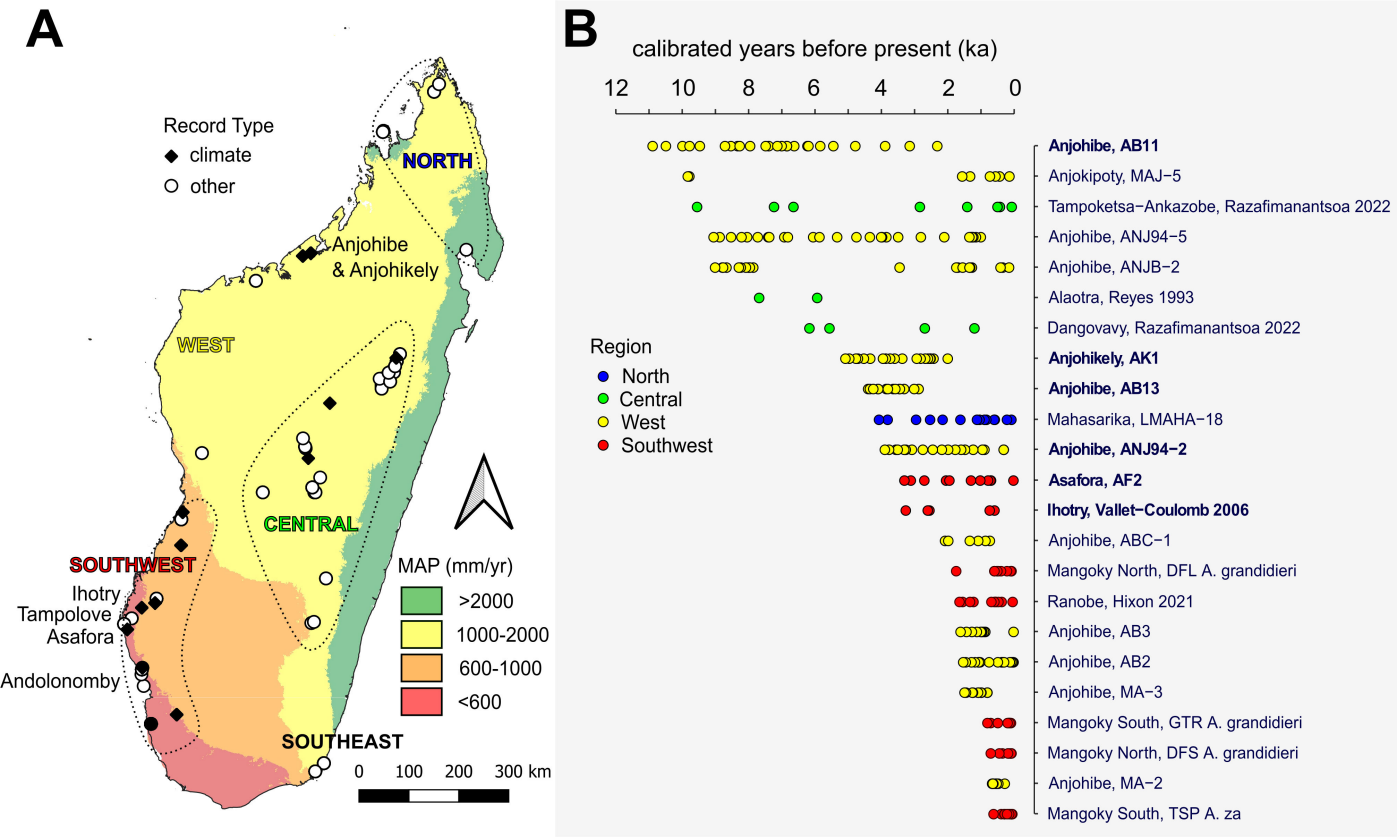
Locations of the study site in southwest Madagascar (Tampolove) and sites with proxy records used to infer past changes in climate and other palaeoenvironmental variables (vegetation, erosion, fire and larger herbivore abundance) (A), and direct dates from different layers of each record used to establish chronologies in age-uncertain records sensitive to climate (B). Geographical groups of sites in A (defined by close proximity and comparable elevation and mean annual precipitation taken from WorldClim 2.1) yield the colour-coded records in B. Names of records discussed in the text are given in bold, consisting of site name and either record collection ID or lead author name and publication year. Note that only age-uncertain records with greater than one direct date during the Holocene are shown in B and that some of these records also include proxy data that are sensitive to vegetation.

Given the distinction between local habitat aridity and regional aridity, the records of each type of aridity must rely on different proxies. On Madagascar, regional records of Holocene climate come primarily from speleothems collected in the southwest and northwest, and few extend beyond the past 4000 years (figure 1). The absence of speleothem growth during the early mid-Holocene in southwest Madagascar is associated with relatively low summer insolation and probably reflects relatively dry conditions [17,24]. The spatial and temporal coverage of Holocene sedimentary archives is higher, but chronological control is typically lower. A relatively arid interval approximately 4200 years ago is recorded regionally in speleothem records ANJ94-5 and AK1 from Anjohibe, northwest Madagascar [25,26] and in a vertebrate death assemblage associated with the salinization of coastal wetlands in Mauritius [27]. Although there is evidence for asynchronous climate change across the island [28], a relatively common feature of records from the island are traces of an arid interval during the Medieval Warm Period approximately 1000 years ago [7,19,28,29], which roughly coincided with the disappearance of multiple large endemic animals on the island [10]. In coastal southwest Madagascar, a decline in relative sea level and coastal water tables could have exacerbated local water scarcity within the last couple of millennia [30].

For decades, researchers have used the stable carbon and nitrogen isotope (*δ*^13^C and *δ*^15^N) content of bone to infer long-term records of diet and habitat (e.g. [31,32]). During an animal’s life, tissues that are slowly remodelled (e.g. bone collagen) act as ecological integrators, essentially averaging over many meals drawn from a potential variety of habitats [33]. The variety of habitats that are integrated depends on a range of species-specific attributes such as body size, locomotor strategy and digestive physiology. Herbivore bone protein *δ*^13^C values reflect primarily the photosynthetic pathway of the plants that they consume [31,34] and to a lesser extent the physiological responses of plants to environmental stressors such as water scarcity [35]. The expected signal in herbivore collagen *δ*^13^C values in southwest Madagascar is well established based on existing C_3_, C_4_ and Crassulacean acid metabolism (CAM) plant datasets, with collagen relatively enriched in ^13^C reflecting greater reliance on C_4_ grasses and CAM succulents [36,37]. This enrichment of collagen in ^13^C is clear in the case of introduced ruminants in southwest Madagascar, which included zebu cattle (*Bos indicus/taurus*), goats (*Capra hircus*) and sheep (*Ovis aries*) [5]. Existing endemic herbivore collagen *δ*^13^C datasets and associated inference have failed to identify vertebrate grazers on the island (e.g. [5,38–40]). While the high N content of C_3_ plants relative to C_4_ plants makes the former attractive to browsers, high-quality forage is not always available, which can explain some of the variation in endemic herbivore collagen *δ*^13^C values. This leaves some potential for direct interactions via foraging between introduced and endemic herbivores.

**Figure 2.**
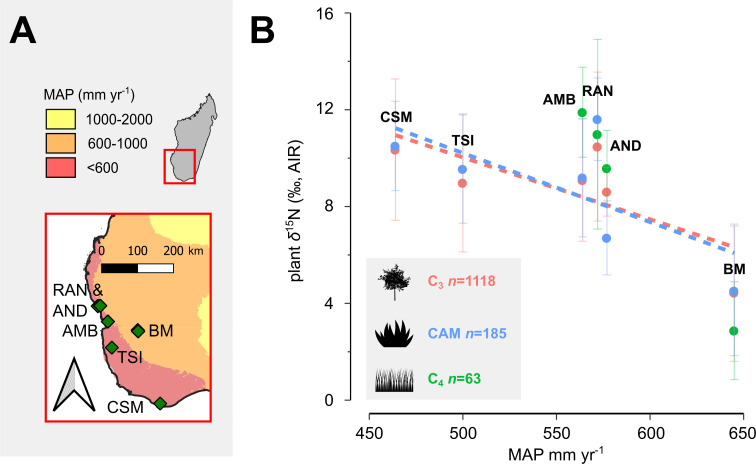
Locations of sites in southwest Madagascar that include previously published *δ*^15^N data from modern plant tissues (*n* = 1366) (A), and plant tissue *δ*^15^N data expressed relative to atmospheric nitrogen (AIR) separated according to photosynthetic pathway and as a function of mean annual precipitation (B). MAP estimates come from WorldClim 2.1, and dashed lines for plant groups with *n* > 100 give linear fits within each group. Abbreviated site names include Beza Mahafaly (BM), Ambohimahavelona (AMB), Andrevo (AND), Ranobe (RAN), Tsimanampesotse (TSI) and Cap Sainte Marie (CSM).

The interpretation of herbivore *δ*^15^N values is context-dependent owing to a multitude of variables that influence soil and plant *δ*^15^N values [41–43]. Herbivore physiology also influences the observed offsets between plant and herbivore collagen *δ*^15^N values, which can complicate direct comparisons of collagen *δ*^15^N between taxa [44]. Generally, relatively open soil nitrogen cycling in arid environments tends to drive higher ecosystem *δ*^15^N values [45,46]. Indeed, across Madagascar, mean annual precipitation (MAP) explains much of the variation in plant and mouse lemur fur *δ*^15^N values, with C_3_ plant *δ*^15^N values tending to decrease approximately 3‰ per 500 mm yr^−1^ increase in MAP [36]. This trend is more pronounced in relatively arid sites and is shared among sympatric C_3_, C_4_ and CAM plants (figure 2; [36,47–49]). However, there are multiple examples in the region of plant *δ*^15^N values that vary by >2‰ independent of MAP and over short distances, according to substrate and proximity to surface water [36,47,48].

Previous attempts to recognize signals of environmental change in collagen *δ*^15^N records have been complicated by the local heterogeneity of primary producer *δ*^15^N values, coupled with both limited site- and taxon-specific sample sizes and limited timespans represented in bone deposits [5,37,50,51]. However, at Tampolove, southwest Madagascar, bones of giant tortoises and pygmy hippos were deposited under rock ledges and in small ponds for several millennia before the local disappearance of these animals [18]. These relatively continuous deposits, which capture a time window extending from approximately 6000 to 1000 years ago in the case of tortoises, thus provide an excellent opportunity to explore how these animals responded to past arid intervals.

I hypothesized that pygmy hippos in the vicinity of Tampolove, unlike giant tortoises, were sensitive to aridification and consistently relied primarily on C_3_ plants in wet habitat even during times of regional climate drying. Consequently, I expected tortoise and hippo collagen *δ*^13^C and *δ*^15^N values to diverge over millennia. I also predicted relatively high variance in giant tortoise collagen *δ*^13^C and *δ*^15^N values and that these stable isotope values would be positively correlated, reflecting greater reliance on C_3_ plants when relatively wet habitat was exploited. Finally, I expected maxima only in the tortoise collagen records to approximately coincide with relatively arid intervals present in regional records, such as those approximately 4200 and 1000 years ago.

## 2. Methods

### (a) Data collection

Purified bone collagen samples from 47 specimens previously described by Hixon *et al*. [18] were submitted to the Yale Analytical and Stable Isotope Center for *δ*^13^C and *δ*^15^N analysis (table 1; electronic supplementary material, dataset S1). To help build comparisons among both introduced and endemic taxa, this analysis included both 36 hippos and tortoises plus 11 samples from other taxa (including seven ruminants, namely zebu and goats). The analysis was completed with a Thermo DeltaPlus Advantage elemental analyzer isotope ratio mass spectrometer, where the long-term precision of *δ*^13^C and *δ*^15^N measurement is 0.2‰ and the accuracy of *δ*^13^C and *δ*^15^N values is 0.1‰ and 0.2‰, respectively. These measurements were combined with data previously published from another 46 specimens collected in the area (thus totalling 93 specimens; table 1 [5,52]). In practice, *δ*^13^C and *δ*^15^N measurements from different laboratories differ, and a review of 21 laboratories (including that at Oxford University, which analysed samples for [52]) found that the average pairwise interlaboratory differences in collagen *δ*^13^C and *δ*^15^N values were 0.2‰ and 0.4‰, respectively [53].

**Table 1.** Summary of sample sizes and chronological spread of samples according to taxon.

taxon^a^	common name	this article	previous pub.	total^b^	span (cal BP)^c^	mean gap (yr)
*Hippopotamus* ^†^	hippo	16	15	31 (27)	4920−945	153
*Aldabrachelys* ^†^	tortoise	20	9	29 (22)	6095−1025	241
*Bos taurus indicus*	zebu	5	15	20 (11)	860–present	227
*Capra hircus*	goat	2	4	6 (0)	na	na
*Ovis* or *Capra*	sheep or goat	0	1	1 (1)	na	na
*Potamochoerus larvatus*	bushpig	0	1	1 (1)	na	na
*Crocodylus*	crocodile	2	0	2 (2)	3570−2400	1170
*Mullerornis* ^†^	elephant bird	1	1	2 (2)	4465−2475	1990
*Aepyornis* or *Mullerornis*^†^	elephant bird	1	0	1 (1)	na	na

^a^ Extinct taxa are marked with (^†^).

^b^ Number in parentheses specifies number directly ^14^C-dated with C : N <3.6 that are not duplicates.

^c^ Span is mean cal BP oldest and youngest.

The protein fraction of subfossil bone is useful for isotopic analysis given that, unlike porous bone mineral, there is limited potential for isotopic exchange between endogenous collagen and the burial environment [54]. However, subfossil collagen samples must be checked for traces of severe degradation and contamination. The presence of exogenous C may affect collagen *δ*^13^C measurements, but the magnitude of this effect depends upon the *δ*^13^C values of both the sample protein and soil contaminants [55]. Severe degradation of endogenous protein through time, resulting in samples with collagen yields <0.5% initial bone mass, may also affect amino acid abundances and thus collagen *δ*^13^C and *δ*^15^N values [56]. Seven ancient samples (four tortoises, two hippos and one zebu) were flagged as having potentially unreliable ^14^C data given that they had relatively high atomic C : N values (3.6−4.3), and two of these had purified collagen yields of <1% initial bone mass (electronic supplementary material, dataset S1 [56]). Note that the relationship between C : N values and preservation of amino acid profiles in bone is generally not one-to-one [57]. In the dataset presented here, the absence of correlations between atomic C : N values and *δ*^13^C and *δ*^15^N values for samples with C : N < 3.6 (electronic supplementary material, figure S1) suggests that variable preservation does not dominate the stable isotope signals in this subset of data. Yet, this observation does not exclude the possibility that some of the *δ*^13^C and *δ*^15^N data from samples with C : N > 3.6 are reliable. I took a conservative approach, and samples with relatively high C : N values were excluded from all analyses given the lack of chronological control and the primary focus of this study on change through time. An additional five samples (three tortoises and two hippos) were excluded as possible duplicates given that they had similar stable isotope data and, in two cases, indistinguishable ^14^C ages (electronic supplementary material, dataset S1). These duplicates follow from both pond sediment mixing and highly fragmentary giant tortoise remains [18].

### (b) Data analysis

To compare δ^13^C data from ancient and modern bone, I corrected the *δ*^13^C of the modern sample values according to the recent depletion of atmospheric CO_2_ in ^13^C (Suess effect, following the approach of [49]). For taxa with *n* > 10 (*Bos, Hippopotamus* and *Aldabrachelys*), I checked for normality in the distribution of *δ*^13^C and *δ*^15^N values using the Shapiro–Wilk test and checked for equal variance in stable isotope values between taxa within each isotope system using the Brown–Forsythe test. Given that these groups passed these tests of normality (*p* ≥ 0.230) and equal variance (*p* ≥ 0.083), I compared group mean *δ*^13^C and *δ*^15^N values through one-way ANOVA and did post hoc pairwise multiple comparisons among *δ*^13^C values through the Holm–Sidak method. I checked for monotonic associations between *δ*^13^C and *δ*^15^N values within taxa using Spearman correlation coefficients. All analyses were completed in R v. 4.3.3.

To help put collagen *δ*^13^C values from well-represented taxa in terms with biological meaning, I used a mixing model to infer the fraction of sample *δ*^13^C signal derived from C_3_ plant material. I followed a conservative approach and assumed a large uncertainty around the expected offset between diet and consumer protein *δ*^13^C values (3.2 ± 2.0‰ [58]). Modern plant *δ*^13^C values, corrected for the Suess effect to be comparable to ancient specimen *δ*^13^C values (electronic supplementary material, dataset S2), formed the basis of mixing model endmembers. Given the regional similarity between C_4_ plant *δ*^13^C values (*n* = 64, *x̄* = −12.6‰, s.d. = 1.1‰) and those of CAM plants (*n* = 185, *x̄* = −13.6‰, s.d. = 1.5‰), I chose to focus on only C_3_ and C_4_ mixing model end members. The modelling was completed in ReSources (https://github.com/Pandora-IsoMemo), which is the updated version of the Bayesian mixing model FRUITS. Diet endmember contributions to each consumer *δ*^13^C value were modelled independently, and the associated model file is included in the electronic supplementary material.

For taxa with relatively good temporal coverage (*Hippopotamus* and *Aldabrachelys*, with mean gaps between directly dated individuals of <250 years and sample sizes of >20 individuals), I created taxon-specific *δ*^13^C and *δ*^15^N records according to time and propagated chronological uncertainty using GeoChronR [59]. The uncertainties associated with the calibrated ^14^C age for each individual are given in the electronic supplementary material, dataset S1. Note that subsequent mentions of the time intervals over which *δ*^13^C and *δ*^15^N records vary consider the chronological uncertainty propagated through GeoChronR, which accounts for non-normal calibrated age distributions. An approximately 600 year chronological gap in the *Bos taurus indicus* record currently prevents meaningful consideration of continuous change in stable isotope values through time for this species.

To identify relevant palaeoclimate records for comparisons, I compiled chronological data from age-uncertain records across Madagascar that include proxies sensitive to past climate or environmental change. This included a search through existing repositories (NOAA, PANGEA, Neotoma and Lipdverse) and an earlier review of chronological data from the island [60]. This yielded a total of 639 absolute age determinations (radiocarbon and uranium thorium) from 77 age-uncertain records collected across the island (electronic supplementary material, dataset S3; figure 1). Although some of these data were published as early as the 1960s, approximately 86% of these age determinations were published only recently (between 2016 and 2024). Excluded from the compilation are incomplete entries, such as ^14^C dates reported only in calibrated years before present. This compilation of age determinations is housed with a pre-existing compilation of stable isotope data from the island in the IsoMad data community on the Pandora data platform (https://pandoradata.earth/organization/isomad-isotopic-data-of-madagascar).

To aid comparisons with the Tampolove animal stable isotope records, I plotted selected palaeoclimate records (bolded in figure 1) and propagated chronological uncertainty through GeoChronR. Corrected U/Th dates were modelled in BChron as normal distributions, ^14^C records were calibrated with SHCal20 in BChron, and ^14^C dates from Asafora were calibrated with SHCal20 in OxCal4.4 before being modelled with U/Th dates as normal distributions in BChron. During analysis, I excluded dates from the Anjohibe ANJ94-2 record (*n* = 3, following [61]), Anjohibe AB11 record (*n* = 4, following [62]) and Anjohikely AK1 record (*n* = 1, following [26]). Note that the Anjohibe ANJ94-2 and Anjohibe AB11 and AB13 records include a combination of calcite and aragonite. In the case of ANJ94-2, aragonite *δ*^18^O values were converted to calcite *δ*^18^O values following Railsback *et al*. [61]. Railsback *et al*. [61] observe five layer-bounding surfaces that suggest hiatuses in CaCO_3_ precipitation, and Scroxton *et al*. [26] note two significant hiatuses. I used the depths of these layers and the age-depth models from BChron to estimate the times of these hiatuses.

## 3. Results

**Figure 3.**
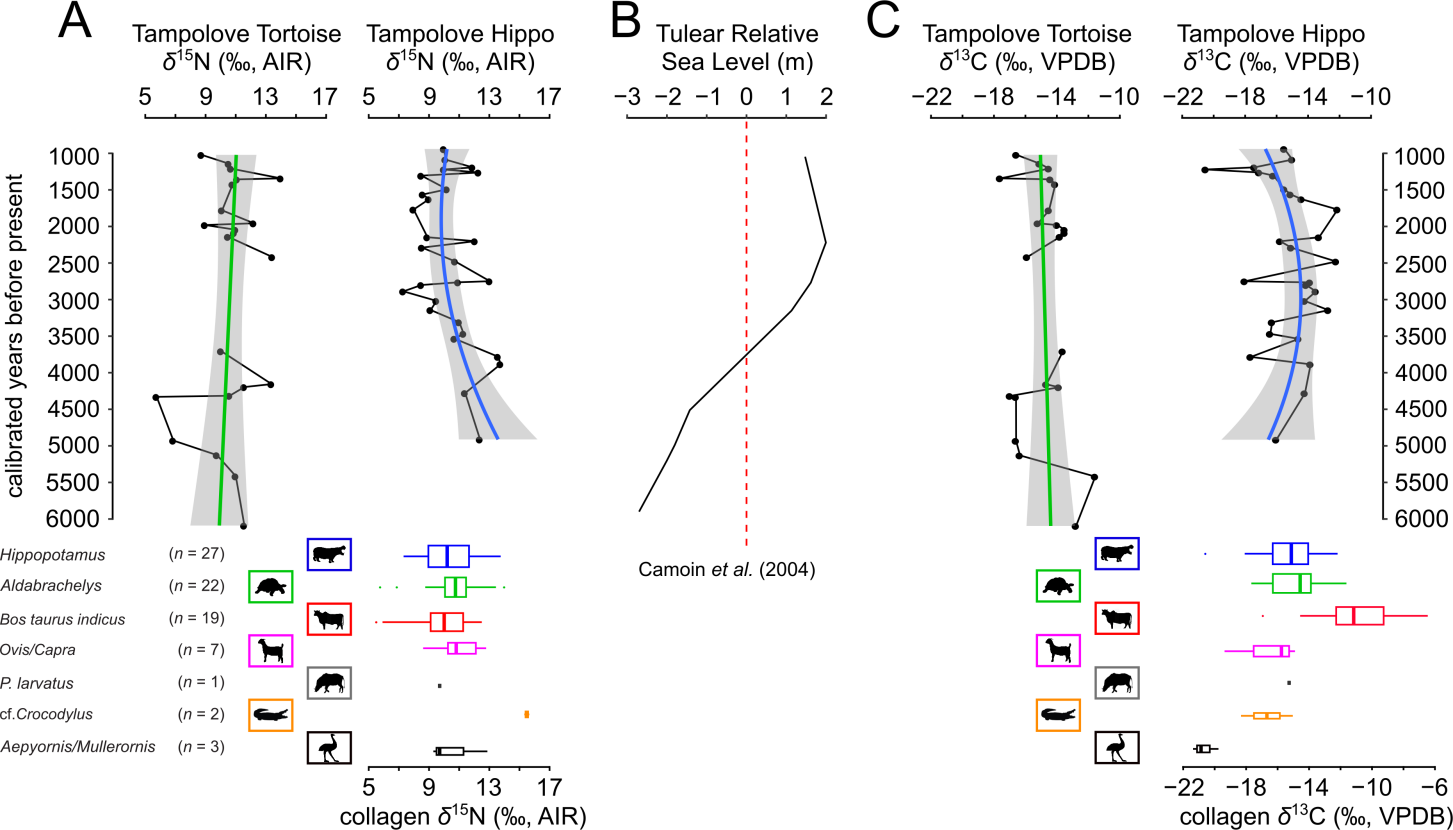
Tampolove animal collagen *δ*^15^N and *δ*^13^C value distributions and changes through time (A,C) plotted against past change in relative sea level (B). The *δ*^15^N and *δ*^13^C values from directly ^14^C-dated tortoise and hippo collagen are marked according to mean calibrated years before present, and broad trends are marked by a linear model to the tortoise data (green) and a second-order polynomial to the hippo data (blue), with the 95% confidence intervals marked with grey shading. Box plots illustrating *δ*^15^N and *δ*^13^C data from the colour-coded taxa have widths scaled to sample size, boxes that illustrate interquartile ranges, and whiskers that extend to minimum/maximum points that fall within 1.5 times the interquartile ranges. The *δ*^13^C data are expressed relative to Vienna Peedee belemnite (VPDB).

Tortoise and hippo collagen *δ*^13^C and *δ*^15^N values have similar distributions but vary substantially through time (figure 3). When considering tortoises, hippos and zebu, there are taxon-specific differences in mean *δ*^13^C values (*F*_2_ = 27.665, *p* < 0.001), and pairwise comparisons suggest that zebu collagen (*δ*^13^C *x̄* ± s.d. = −10.9 ± 2.7‰) is significantly enriched in ^13^C relative to that of pygmy hippos and giant tortoises (*δ*^13^C of −15.2 ± 1.9‰ and −14.8 ± 1.5‰, respectively). The *δ*^13^C mixing model results suggest that, on average, 40–50% of tortoise and hippo protein C came from C_3_ plant material (electronic supplementary material, dataset S1). In a couple of extreme cases (with tortoise and hippo collagen *δ*^13^C values of approximately −12‰), over 80% of tortoise and hippo protein C could have come from C_4_ or CAM plant material.

There is no significant difference in *δ*^15^N values among tortoises, hippos and zebu (*F*_2_ = 0.927, *p* = 0.40; figure 3). However, the covariance between collagen *δ*^13^C and *δ*^15^N values differs among taxa. Hippos include the only significant association between collagen *δ*^13^C and *δ*^15^N values, and it is negative (electronic supplementary material, figure S2; figure 3A,C; *r*_s_ = −0.48, *p* = 0.01, *n* = 27). Tortoise and hippo *δ*^13^C values have very similar interquartile ranges (−16.4 to −13.8‰ and −16.3 to −13.9‰, respectively), but there is no hint of a negative relationship between tortoise collagen *δ*^13^C and *δ*^15^N values (electronic supplementary material, figure S2; *r*_s_ = 0.18, *p* = 0.41, *n* = 22). Similar to hippos, zebu collagen *δ*^13^C and *δ*^15^N values are negatively correlated, but, in the case of zebu, this association is not statistically significant (*r*_s_ = −0.22, *p* = 0.36, *n* = 19).

Unlike other continuous records from sediments or speleothems, animal stable isotope records have each measurement necessarily anchored in time, but their temporal resolution is limited by the distribution of bone ages. The average time separating the mean calibrated age of tortoise specimens (241 years, table 1) is greater than that for hippos (153 years), but the average spacing of tortoises drops to 189 years when omitting an exceptionally large gap of 1287 years during 3711−2424 cal BP (figure 3). The difference in *δ*^15^N values between tortoises and hippos apparently but insignificantly varies through time (figure 3A). This follows from a general decline in hippo *δ*^15^N values during 5000−1000 cal BP, during which relative sea level in nearby Tulear, southwest Madagascar, surpassed present levels approximately 3500 cal BP and reached a maximum approximately 2500 cal BP (figure 3B; [63]). Specifically, the mean hippo collagen *δ*^15^N value during 4920−3710 cal BP (*n* = 4, 12.8 ± 1.1‰) is apparently but insignificantly greater than that of contemporary tortoises (*n* = 5, 10.3 ± 1.1‰ *t*_7_ = 1.668, two-tailed *p* = 0.139). This tendency is reversed after the tortoise record resumed in 2425 cal BP, with the mean hippo collagen *δ*^15^N value (*n* = 13, 9.8 ± 1.5‰) apparently lower than that of contemporary tortoises (*n* = 13, 11.0 ± 1.5‰, *t*_24_ = 1.974, two-tailed *p* = 0.06).

**Figure 4.**
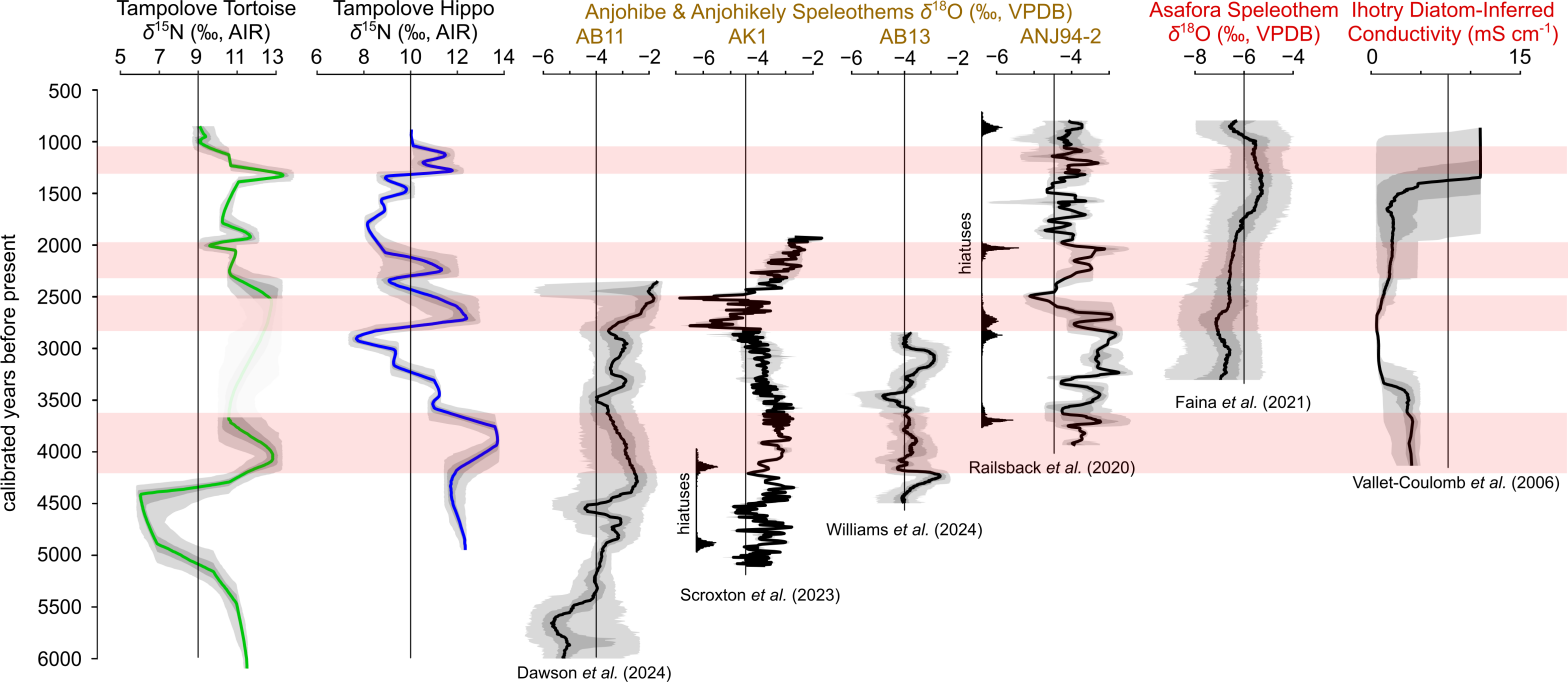
Tampolove tortoise and hippo collagen *δ*^15^N values plotted against time and relative to regional palaeoclimate records from northwest Madagascar (brown) and southwest Madagascar (red). Chronological uncertainty is propagated using GeoChronR, and grey ribbons mark quantiles around proxy estimates given by black lines in each case aside from the tortoise and hippo *δ*^15^N records (green and blue, respectively). Age–depth models are based on U/Th dates (Anjohibe/Anjohikely records), ^14^C dates (Tampolove and Ihotry records) and a combination of U/Th and ^14^C data (Asafora record). Solid distributions associated with AK1 and ANJ94−2 mark the modelled distributions for intervals that include hiatuses in these records. Red horizontal shading marks intervals with maxima in at least hippo collagen *δ*^15^N values that are discussed in the text.

After propagating chronological uncertainty, maxima in hippo and tortoise collagen *δ*^15^N values during approximately 4200–3800 cal BP and 1500–1200 cal BP roughly coincide with each other and with arid intervals in regional records (figure 4). The most prominent peaks in hippo and tortoise collagen *δ*^15^N values (including the two highest for hippos and second highest for tortoises) roughly coincide during approximately 4200–3800 cal BP and follow a pronounced minimum in tortoise *δ*^13^C and *δ*^15^N values (figure 4). Regional speleothem and lake sediment records suggest that at least some of this interval was relatively arid. Specifically, a sharp increase in speleothem *δ*^18^O values at Anjohibe, approximately 4200 cal BP [64] reflects relatively light precipitation events during this time. At least two other Anjohibe speleothem records from this interval include hiatuses, which follow from the temporary absence of drip water [25,26]. Continuous palaeoclimate records from southwest Madagascar during this interval are limited to a lake sediment record from Lac Ihotry. The changing ecological requirements of dominant diatoms present in Lac Ihotry sediments suggest that the water in this lake had greater conductivity, was probably relatively salty, and experienced a higher ratio of evaporation to precipitation during approximately 4000–3500 cal BP than during the following millennium [30].

Relatively minor peaks in hippos and tortoise collagen *δ*^15^N values (nonetheless including the highest tortoise *δ*^15^N value) roughly coincide during approximately 1500–1200 cal BP (figure 4). Although chronological resolution in most records from southwest Madagascar is low during this time, it was during approximately 1500–1000 cal BP that relative sea level declined [63], a speleothem *δ*^18^O record from Asafora (<15 km distant from Tampolove) includes an extended maximum [28], and water tables lowered in coastal ponds around Tampolove and in Lac Ihotry [18,30]. Significantly, three hippo collagen *δ*^15^N values and two tortoise *δ*^15^N values document decreasing trends in the following two centuries approximately 1200–1000 cal BP, which immediately precede the local disappearance of these animals. Although Asafora *δ*^18^O values remain high during most of this interval [28] and an ostracod *δ*^18^O record from Ranobe may document a drying trend during these centuries [19], it was during approximately 1200–1000 cal BP that lacustrine sediment most recently started to fill Namonte Basin (approx. 20 km from Tampolove [65]).

During the extended gap in the tortoise bone record, a pronounced peak in hippo collagen *δ*^15^N is defined by six values during approximately 2900–2300 cal BP (figure 4). This roughly coincides with increases in Anjohibe speleothem *δ*^18^O values [26,62] and hiatuses in at least one speleothem from this site [61]. Relative sea level was near its maximum during this interval (+2 m relative to present [63]), and the record of diatom-inferred conductivity from Lac Ihotry includes no positive excursion during this time [30]. The subsequent and smaller maximum in the hippo collagen *δ*^15^N record approximately 2200 cal BP is defined by a single measurement and is not matched by a relatively high *δ*^15^N value in a tortoise that died at approximately the same time.

## 4. Discussion

Based on the hypothesis that hippos were drought-sensitive relative to giant tortoises, I predicted that stable isotope records of these animals around Tampolove diverged during the mid to late Holocene as tortoises tracked fluctuations in regional aridity and hippos consistently exploited mesic habitat. Counter to expectation, I found generally similar distributions in hippo and tortoise collagen *δ*^13^C and *δ*^15^N values. Although taxon-specific *δ*^13^C and *δ*^15^N records covaried in different ways, both hippo and tortoise *δ*^15^N values document exploitation of dry habitat during what were probably relatively arid times. These results have important implications for the inference of past extinction drivers and for understanding regional palaeoclimate.

Differences in the covariance of hippo and tortoise collagen *δ*^13^C and *δ*^15^N values suggest that these groups responded differently to regional changes in moisture availability. The negative relationship between hippo collagen *δ*^13^C and *δ*^15^N values suggests that, when hippos consumed plants in relatively mesic patches, they tended to prefer more C_4_ or CAM plants. This is consistent with the observation of Crowley *et al*. [37], who further argue that the consumption of C_4_ grasses by hippos is most parsimonious given the xeric preference of CAM plants in the region. Given this assumption, and the fact that C_3_ plants are more digestible [66], it is noteworthy that the majority of protein C for hippos around Tampolove sometimes came from C_4_ grasses. This preference made hippos more likely to interact with introduced grazers and pastoralists [5,19], particularly during relatively mesic intervals. Unlike hippo collagen *δ*^13^C and *δ*^15^N values, those of tortoises have a positive (yet statistically insignificant) association, which may suggest some preference for C_3_ plant material in relatively mesic habitat. These divergent tendencies are important to consider when evaluating synergy hypotheses for extinction that invoke introduced species, forest clearance and aridification.

Coincident fluctuations in regional palaeoclimate records and hippo and tortoise collagen *δ*^15^N records suggest that these records are little affected by a behavioural filter. In other words, although hippos and tortoises preferred variable combinations of plant types, these animals were to some extent flexible and able to track changes in water availability during the mid- to late Holocene. Consequently, their isotopic records can be interpreted primarily in terms of past environmental change.

The prominent peaks in hippo and tortoise collagen *δ*^15^N values 4200−3800 cal BP suggest that the ‘4.2 ka event’ of climate drying affected southwest Madagascar. This is noteworthy given that the geographical extent and mechanism for this drying trend remain uncertain [26,62,67]. Although mid-Holocene subfossil records in southwest Madagascar are scarce, they may be among the few that are locally available given contemporary hiatuses in sedimentary and speleothem records [24,68]. However, as in northwest Madagascar, the severity of this dry interval around Tampolove remains unclear. On Mauritius (approx. 920 km east of Madagascar), a contemporary massive death assemblage of a diverse suite of vertebrates, including dodos (*Raphus cucullatus*) and giant tortoises (*Cylindraspis* spp.), is associated with surface water scarcity and salinization and suggests that the ‘4.2 ka event’ on this island was severe [27]. Such a deposit has not been recovered in coastal southwest Madagascar, possibly because climate drying here was relatively subtle or because endemic vertebrates in southwest Madagascar were less sensitive to water scarcity than those on Mauritius.

The last time interval that includes synchronous peaks in hippo and tortoise collagen *δ*^15^N values approximately 1500–1200 cal BP is much better represented in a diversity of palaeoenvironmental records that are located within southwest Madagascar [19,28,30,65]. However, these *δ*^15^N peaks are less pronounced than those associated with the ‘4.2 ka event’ and those in the hippo record approximately 2900–2300 cal BP. This suggests that both hippos and tortoises had persisted locally during intervals before 1500 cal BP that may have been drier for longer. This interpretation, coupled with the subsequent declines in hippo and tortoise *δ*^15^N values and the local extirpation of these animals during what may have been a trend towards increasingly mesic conditions [18], makes it difficult to explain the disappearance of these animals as a consequence of climate drying. If anything, introduced livestock may have been relatively sensitive to water scarcity [5]. Consequently, it is essential that future tests of synergy hypotheses involving climate drying and the spread of introduced species assess how the potential antagonists of extinct endemic vertebrates probably responded to water scarcity.

Preservation sets a fundamental limit on inference from the subfossil record, and this is clear in the taxonomic resolution, sample size and temporal coverage of the present study. In the absence of DNA analysis, the taxonomic resolution is necessarily limited to genus. However, multiple extinct species from the region have been described within *Hippopotamus* [69] and *Aldabrachelys* [70], and they may have had distinct habitat and dietary preferences [69,71]. These preferences may affect the patterns in figure 3 in ways that are currently unknown and thus complicate inference of palaeoenvironment from bone stable isotope records. Although the temporal resolution of the present study is substantial compared to that at many other subfossil sites from the island, the >1000 year gap in the tortoise bone record prevents inference of whether these animals also lived in arid habitat during approximately 2900–2300 cal BP. This gap is probably a product of taphonomy and could be filled through further field sampling efforts in the karst around the study site. The absence of tortoises, at least from coastal ponds around Tampolove during this interval, may be explained by (i) the tendency for tortoise bone to accumulate around rock ledges in open air, and (ii) the rise in relative sea level above present levels approximately 3500 cal BP [63], which coincided with the filling of coastal ponds in the area [18].

Despite complications in their creation and interpretation, long-term stable isotope datasets from the biological remains of specific taxa can give valuable insight into both regional climate change and biotic responses to this change [22,41,72]. Insight on the particular habits of extinct animals raises new questions [73] and can help refine synergy hypotheses for past extinctions. These hypotheses give useful context to modern studies that seek to identify interactions among enduring threats to endemic biodiversity such as hunting, introduced antagonists, forest clearance and climate change.

## Data Availability

All data are available in the electronic supplementary material, and the data compilation in the electronic supplementary material, dataset S3 is maintained on the Pandora data platform. Supplementary material is available online [74].
